# *PNPLA3* genetic variants determine hepatic steatosis in non-obese chronic hepatitis C patients

**DOI:** 10.1038/srep11901

**Published:** 2015-07-03

**Authors:** Chung-Feng Huang, Jyh-Jou Chen, Ming-Lun Yeh, Ching-I Huang, Ming-Yen Hsieh, Hua-Ling Yang, Chia-Yen Dai, Jee-Fu Huang, Zu-Yau Lin, Shinn-Cherng Chen, Wan-Long Chuang, Yao-Li Chen, Ming-Lung Yu

**Affiliations:** 1 Graguate Institute of Clinical Medicine, College of Medicine, Kaohsiung Medical University, Kaohsiung, Taiwan; 2 Hepatobiliary Division, Department of Internal Medicine, Kaohsiung Medical University Hospital, Kaohsiung, Taiwan; 3 Department of Occupational Medicine, Kaohsiung Municipal Ta-Tung Hospital, Kaohsiung Medical University Hospital, Kaohsiung Medical University, Kaohsiung, Taiwan; 4 Faculty of Internal Medicine, School of Medicine, College of Medicine, and Center for Lipid and Glycomedicine Research, Kaohsiung Medical University, Kaohsiung, Taiwan; 5 Division of Gastroenterology and Hepatology, Chi-Mei Medical Center, Liouying, Tainan; 6 Graduate Institute of Clinical Medicine, College of Medicine, Kaohsiung Medical University, Kaohsiung, Taiwan; 7 Department of Internal Medicine, Kaohsiung Municipal Hsiao-Kang Hospital, Kaohsiung Medical University Hospital, Kaohsiung Medical University, Kaohsiung, Taiwan; 8 Department of Internal Medicine, Kaohsiung Municipal Ta-Tung Hospital, Kaohsiung Medical University Hospital, Kaohsiung Medical University, Kaohsiung, Taiwan; 9 Department of Preventive Medicine, Kaohsiung Medical University Hospital, Kaohsiung, Taiwan; 10 Division of General Surgery, Department of Surgery, Changhua Christian Hospital, Changhua, Taiwan; 11 Institute of Biomedical Sciences, National Sun Yat-Sen University.

## Abstract

The influence of patatin-like phospholipase domain-containing 3 (*PNPLA3*) genetic variants in the development of liver steatosis in Asian chronic hepatitis C patients remains elusive. A total of 1018 biopsy-proven chronic hepatitis C patients were enrolled for evaluation. The proportions of *PNPLA3* rs738409 GG genotype carriage were 7.8% (44/563), 15.8% (58/367) and 19.3% (17/88) in patients with no (liver fat content <5%), mild (5–33%) and moderate/severe (>66%) hepatic steatosis, respectively (trend P < 0.001). Stepwise logistic regression analysis revealed that the strongest factor independently associated with steatosis was the carriage of the *PNPLA3* rs738409 GG genotype (odds ratio [OR]/95% confidence intervals [CI]:2.34/1.557–3.515, P < 0.001). Among the patients with BMI < 24 kg/m^2^, carriage of the rs738409 GG genotype was the only factor associated with hepatic steatosis (OR/CI:3.44/1.824–6.500, P < 0.001). *PNPLA3* genetic variants had minimal effects on hepatic steatosis among overweight or obese patients. Compared to patients with BMI<24 kg/m^2^/non-GG genotype, those with BMI>24 kg/m^2^/GG genotype were more likely to have hepatic steatosis (OR/CI:3.87/2.292–6.524, P < 0.001). In conclusions, both *PNPLA3* genetic variants and BMI played important roles in hepatic steatosis among Asian chronic hepatitis C patients. However, the genetic effect was mainly restricted to non-obese patients.

Hepatic steatosis is more frequently observed in patients with chronic hepatitis C virus (CHC) infection than in the general population. The frequency of significant steatosis in CHC patients who carry variable attributive factors ranges between 40% and 80%, which is approximately twofold higher than in the general population^1^,^2^,^3^,^4^. The presence of hepatic steatosis may promote liver fibrosis progression in the natural course of CHC^5^. In addition, it may also determine the treatment efficacy of interferon based anti-viral therapy^6^. Two major mechanisms account for the high prevalence of hepatic steatosis in CHC, which include the direct cytopathic effect of the hepatitis C virus genotype 3 (HCV-3) viral protein and the indirect effect of metabolic derangement in patients with HCV non-3 infection^3^,^7^,^8^.

Beyond the issue of virological and environmental factors, host genomes also play a role in hepatic steatosis. Genome-wide association study (GWAS) has demonstrated that a single nucleotide polymorphism (SNP) of patatin-like phospholipase domain-containing 3 (*PNPLA3*) gene was associated with nonalcoholic fatty liver disease (NAFLD)^9^. In addition, *PNPLA3* genetic variants have also been shown to be associated with hepatic steatosis in CHC patients^10^,^11^,^12^. However, the result was not consistent in different cohorts^13^. This finding raised the question of whether the association between *PNPLA3* SNP and hepatic steatosis in CHC patients varies across ethnicities. Meanwhile, it has been reported that the determination of PNPLA3 SNPs in NAFLD was not universal in the same population with different metabolic profiles^14^. Taken collectively, the impact of *PNPLA3* genetic variants on liver steatosis in Asian CHC patients with different characteristics has never been studied. We herein recruited a large CHC cohort with histologically proven steatosis and well-characterized demographics, and we aimed to determine the association of *PNPLA3* genetic variants with hepatic steatosis in an Asian CHC population. Importantly, we also sought to determine whether the influence of this gene differs among subpopulations with different characteristics.

## Methods

A total of 1,018 CHC patients who received pre-antiviral evaluation were consecutively recruited in a medical center and two core regional hospitals in Taiwan from 2001 to 2013. Anti–HCV antibodies were detected using a third-generation, commercially available enzyme-linked immunosorbent assay kit (AxSYM 3.0, Abbott Laboratories, Chicago, IL, USA). Serum HCV RNA was detected using qualitative real-time polymerase chain reaction (PCR) (COBAS AMPLICOR Hepatitis C Virus Test, ver. 2.0; Roche, Branchburg, NJ, USA, detection limit: 50 IU/ml) and quantification branched DNA assay (Versant HCV RNA 3.0, Bayer, Tarrytown, New Jersey, USA; quantification limit: 615 IU/ml) before 2011. The HCV genotypes were determined using the Okamoto method before 2011^15^. Both the HCV RNA and genotype were detected using real-time PCR assay (RealTime HCV; Abbott Molecular, Des Plaines IL, USA; detection limit: 12 IU/ml) since 2011^16^. All of the patients received liver biopsies before initiating antiviral therapy. Patients with current or past history of alcohol abuse (≥20 g daily) were excluded in the current cohort. The liver histology was graded and staged according to the scoring system described by Knodell and Scheuer^17^. Hepatic steatosis was evaluated with an H&E stain with the definition of no (liver fat content <5%), mild (5–33%), moderate (33–66%) and severe (>66%) hepatic steatosis. The diagnosis of diabetes was based on 1) laboratory tests with twice the fasting plasma glucose levels *>*126 mg/dL or hemoglobulin A_1_C > 6.5%, or 2) medical history of previously established diagnosis of diabetes. All patients gave written informed consent before enrollment. The study was approved by the ethics committee of Kaohsiung Medical University Hospital and was performed according to the guidelines of the International Conference on Harmonization for Good Clinical Practice.

### *PNPLA3* genotyping

The *PNPLA3* rs738409 was selected as the candidate SNP, and the genotype was determined using the methods described previously^18^.

### Statistical analyses

The frequency was compared between groups using the χ^2^ test with the Yates correction or Fisher’s exact test. Group means, presented as the mean values and standard deviations, were compared using the analysis of variance and Student’s *t* test or the Mann-Whitney U test. The serum HCV RNA levels were expressed after logarithmic transformation of the original values. The influence of *PNPLA3* in liver steatosis was calculated using dominant (genotype CC vs. CG + GG) and recessive (genotype GG vs. CG + CC) genetic models of inheritance. A stepwise logistic regression analysis was performed to evaluate the independent factors associated with steatosis by analyzing the co-variants with P values <0.05 in the univariate analysis. The statistical analyses were performed using the SPSS 12.0 statistical package (SPSS, Chicago, IL, USA). All statistical analyses were based on two-tailed hypothesis tests with a significance level of p < 0.05.

## Results

### Patients

The mean age of the patients included in the study population was 51.8 of years and 56.6% of the patients were male (Table 1). The majority of the patients weighed between 18.5 kg/m^2^ and 30 kg/m^2^. Hepatic steatosis was observed in 455 (44.7%) of the CHC patients. The proportion of no, mild, moderate and severe hepatic steatosis was 55.3% (n = 563), 36.1% (n = 367), 7.8% (n = 79) and 0.9% (n = 9), respectively. *PNPLA3* rs738409 CC, CG, and GG genotypes accounted for 41.5%, 46.9% and 11.7% of the population, respectively. The majority of patients were infected with HCV-1 and HCV-2, whereas only one patient in the current cohort was infected with HCV-3.

### Factors associated with hepatic steatosis in CHC patients

In the univariate analysis, the factors associated with fatty liver included older age, high body mass index (BMI), the presence of diabetes, a high *r-*glutamyltransferase (*r*-GT) level and carriage of the *PNPLA3* rs738409 GG genotype, using the recessive model. CHC patients with hepatic steatosis had numerically higher proportions of CG/GG genotype carriage when compared to those without, as determined by using the dominant model (P = 0.08). Six-hundred and ninety four patients had available cholesterol and triglyceride (TG) data. Patients with steatosis had significantly higher TG level (109 + 64 mg/dL vs. 96 + 49 mg/dL, P = 0.004). Stepwise logistic regression analysis was performed to evaluate factors independently associated with hepatic steatosis. If the variable of TG was not taken into consideration, the strongest factor independently associated with steatosis was carriage of the *PNPLA3* rs738409 GG genotype (odds ratio [OR]/95% confidence intervals [CI]:2.34/1.557–3.515, P < 0.001), followed by body mass index (BMI, OR/CI: 1.12/1.082–1.167, P < 0.001) and age (OR/CI:1.02/1.004–1.028, P = 0.007) by using the recessive model. By using the dominant model, the factors associated with steatosis included carriage of the *PNPLA3* rs738409 CG/GG genotype (OR/CI:1.31/1.013–1.703, P = 0.04), BMI (OR/CI: 1.13/1.084–1.169, P < 0.001) and age (OR/CI:1.02/1.004–1.028, P = 0.006). If the variable of TG was taken into account, the strongest factor associated with liver steatosis remains the carriage of *PNPLA3* rs738409 GG genotype (OR/CI: 2.37/1.408-3.983, P = 0.001) (Table 2). The *PNPLA3* genotype distribution did not differ between patients with or without available TG level either by recessive model (GG genotype: 11.1% vs. 13.0%, P = 0.39) or dominant model (CG/GG genotype: 58.8% vs. 58.0%, P = 0.82).

### Role of *PNPLA3* genetic variants in determining hepatic steatosis among patients with different BMIs

**Figure 1:**
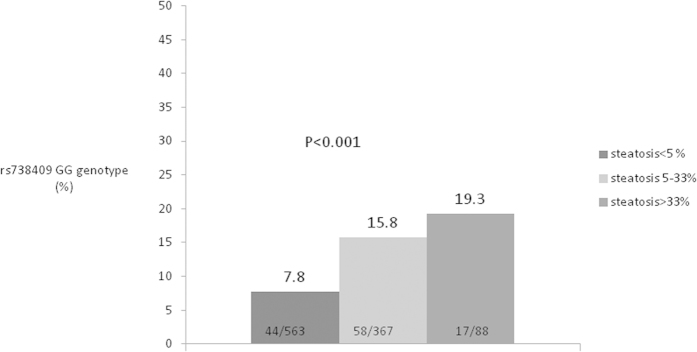
Percentage of *PNPLA3* rs38409 genotype in patients with hepatic steatosis <5%, 5–33% and >33%, respectively.

Because BMI and *PNPLA3* genetic variants are both important determinants of hepatic steatosis, we further explored the influence of the *PNPLA3* SNP in steatosis among patients with different BMIs. Patients were categorized into normal or underweight (<24 kg/m^2^), overweight (24–27 kg/m^2^) or obese (>27 kg/m^2^) according to the definition of the Health Promotion Administration of the Ministry of Health and Welfare in Taiwan^19^. Among the patients with normal body weights, hepatic steatosis was associated with a higher *r*-GT level, a lower p*r*oportion of HBsAg seropositivity and a higher proportion of *PNPLA3* rs738409 G genotype carriage in univariate analysis (Table 3). In multivariateanalysis, carriage of the rs738409 GG genotype was the only factor associated with hepatic steatosis (OR/CI:3.44/1.824–6.500, P < 0.001) by using the recessive model, whereas factors associated with steatosis were the rs738409 GG/GC genotype (OR/CI:1.69/1.101–2.614, P = 0.02) and HBV dual infection (OR/CI: 0.42/0.188–0.940, P = 0.04) using the dominant model (Table 4). Among the overweight patients (BMIbetween 24–27 kg/m^2^), the univariate analysis revealed that the patients with steatosis were older and were more likely to have diabetes, lower platelet counts and a higher *r*-GT level; while the steatotic patients had a substantially higher proportion of rs738409 GG genotype carriage, the difference was not significant (P=0.06) (Table 3). Multivariate analysis revealed that the factors associated with steatosis in overweight CHC patients included age (OR/CI:1.023/1.001–1.045, P = 0.04) and diabetes (OR/CI:2.201/1.055–3.875, P = 0.03), but not *PNPLA3* rs738409 genotype variants (Table 4). For obese patients, hepatic steatosis was only associated with a higher *r*-GT level in the univariate analysis (Table 3), although no factors were associated with hepatic steatosis in obese CHC patients in the multivariate analysis. The impact of the *PNPLA3* rs738409 genotype on fatty liver varied among patients with different BMIs (Supplementary Fig 1). We further explored the role of the SNP in patients with different degrees of hepatic steatosis. The proportion of *PNPLA3* rs738409 GG genotype carriage was 7.8% (44/563), 15.8% (58/367) and 19.3% (17/88) in patients with no, mild, and moderate/severe hepatic steatosis, respectively (trend P < 0.001) (fig. 1).

### Interaction of *PNPLA*3 rs738409 genotype and BMI in hepatic steatosis

**Figure 2:**
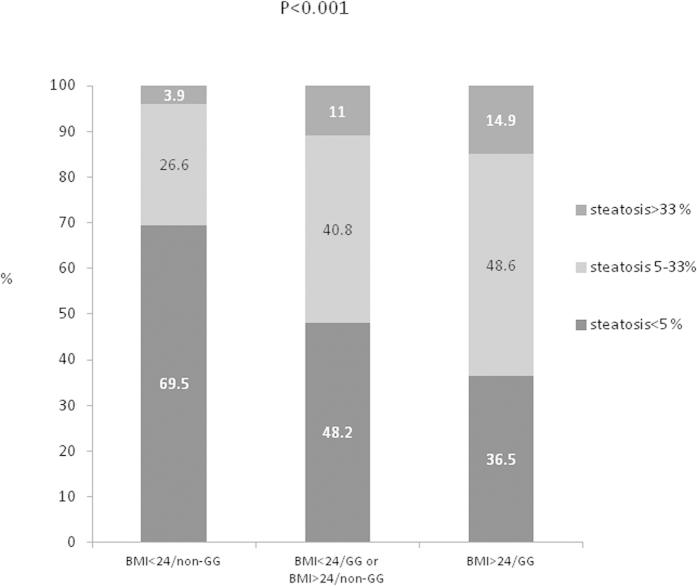
Percentage of patients with hepatic steatosis <5%, 5–33% and >33%, respectively, stratified by body mass index and PNPLA3 rs38409 genotype. Supplementary Figure 1 Proportion of hepatic steatosis in patients with different *PNPLA3* rs38409 genotypes, stratified by BMI.

Both the *PNPLA3* genetic variants and BMI determined hepatic steatosis. We further analyzed their interactions in contributing to fatty liver. There was a significantly increased proportion of mild and moderate/severe hepatic steatosis in patients with BMI>24 kg/m^2^ and the *PNPLA3* rs738409 GG genotype compared to those with BMI<24 kg/m^2^ and/or non-GG genotype (P < 0.001) (fig. 2). The proportion of hepaticsteatosis was 30.5%, 51.8% and 63.5% in patients with BMI<24 kg/m^2^/non-GG genotype, BMI<24 kg/m^2^/GG genotype or BMI>24 kg/m^2^/non-GG genotype, and BMI>24 kg/m^2^/GG genotype, respectively. Compared to patients with BMI<24 kg/m^2^/non-GG genotype, those with BMI>24 kg/m^2^/GG genotype were more likely to have hepatic steatosis (OR/CI:3.87/2.292–6.524, P < 0.001), followed by those patients with BMI<24 kg/m^2^/GG genotype or BMI>24 kg/m^2^/non-GG genotype (OR/CI:2.43/1.849–3.202, P < 0.001) (Table 5).

## Discussion

In the current large-scale study, we demonstrated that the influence of the *PNPLA3* genetic variants in hepatic steatosis remains consistent in Asian CHC populations, and the effect is independent of other metabolic disorders. The association was particularly enhanced through the use of the recessive model. CHC patents who carried the *PNPLA3* rs738409 GG genotype had a 2.3-fold risk of developing hepatic steatosis when compared to their counterparts. Both *PNPLA3* genetic variants and BMI played important roles in hepatic steatosis in CHC patients. Importantly, we identified that the host genetic effect was mainly restricted to non-obese patients and not obese patients. For patients with BMI < 24 kg/m^2^, the carriage of the *PNPLA3* rs738409 GG genotype increased the risk of hepatic steatosis 3.4-fold when compared to those individuals carrying the C allele.

PNPLA3 participates in the restoration of lipid homeostasis upon aberrant intracellular lipid accumulation. The determination of the role of the *PNPLA3* SNP in NAFLD was established in 2008^9^. Later, several reports have linked the genetic variants to other spectrums of liver disease such as HBV^20^ and HCV infection^3^,^7^,^8^,^21^. Due to the direct steatotogenic effect of the HCV-3 protein, the determination of the host genome in hepatic steatosis has been restricted to non-HCV-3 infection^12^,^14^. Most of the studies have originated from the West, where the genetic effect has been fully explored in Caucasians. Nevertheless, Nakamura *et al.* recently reported that there was no association between the *PNPLA3* rs738409 genotype and fatty changes in sonography in Japanese patients with HCV-1 and HCV-2 infections^13^. It is therefore imperative to validate the effect of PNPLA3 in another cohort with different ethnicities and patient characteristics in which the phenotype is clearly defined by liver biopsy. Approximately half of the CHC patients had hepatic steatosis in the current cohort, which was similar to the prevalence in some Western reports^1^,^2^,^3^. However, the mean BMI was only 24.9 kg/m^2^ and less than one tenth of the patients had hepatic steatosis >33% in the current population. We confirmed that the *PNPLA3* genetic variants consistently play a role in hepatic steatosis in Asian patients with HCV-1 and HCV-2 infections. The determined power was similar to the reports from the West where patients homozygous for the risk G allele had an approximately 2-fold higher risk for hepatic steatosis^12^,^21^

As HCV infection increases the risk for liver steatosis and metabolic derangement, the relationship between HBV infection and hepatic steatosis remains conflicting^22^,^23^. We identified that lean CHC patients with HBV co-infection had a lower proportion of liver steatosis compared to those with HCV mono-infection. The finding was in agreement with some reports that HBV infection protects against fatty liver rather than promoting it^23^. This finding may be attributed to a lower frequency of metabolic disorders in HBV carriers^23^. In addition, hepatic steatosis may accelerate hepatitis B surface antigen clearance^24^,^25^. Whether the mechanism supports the inverse relationship between HBV infection and steatosis awaits further confirmation. Hepatic steatosis may promote liver fibrosis progression, although we did not observe this association in the current study. Because fibrotic tissue may replace liver fat content as the disease progresses, the linkage might be masked from cross-sectional observation^18^,^26^.

Interestingly, we observed that the role of the *PNPLA3* genetic variants in hepatic steatosis was particularly enhanced in non-obese patients. The odds ratios further increased compared with those in the whole population, and the *PNPLA3* genotype was the strongest predictor for hepatic steatosis in non-obese subjects. The reason why some lean CHC patients developed fatty liver was not fully understood. The current study in part provided a clue from the perspective of the host genetic profile. . In contrast, the *PNPLA3* SNP did not independently determine hepatic steatosis in overweight or obese patients. The higher the BMI, the less the genetic composition impacted the degree of hepatic steatosis. The current study focusing on CHC patients may echo the report that the *PNPLA3* rs738409 GG genotype increases the risk of NAFLD in the general population without metabolic disorder^14^.

The current study was limited by the absence of metabolic profiles, which may interfere with the final results. Nevertheless, we demonstrated the associations between the *PNPLA3* SNP and other simple demographic characteristics with hepatic steatosis. These findings should facilitate more direct clinical relevance in the interpretation of the association.

In conclusion, we demonstrated that the *PNPLA3* genetic variants determined the risk of development of hepatic steatosis in Asian CHC patients. However, the effect was not universal and was mainly restricted to non-obese patients. Whether the latter finding is generalizable to other ethnicities awaits further validation.

## Additional Information

**How to cite this article**: Huang, C.-F. *et al.* *PNPLA3* genetic variants determine hepatic steatosis in non-obese chronic hepatitis C patients. *Sci. Rep.* **5**, 11901; doi: 10.1038/srep11901 (2015).

## Supplementary Material

Supplementary Information

## Figures and Tables

**Table 1:** Univariate analysis of factors associated with hepatic steatosis.

	All patients(N = 1018)	Steatosis (−) (n = 563)	Steatosis (+) (n = 455)	P value
Age (years, mean + SD)	51.8 + 11.3	50.9 + 11.8	53.0 + 10.6	0.004
Male gender, n (%)	576 (56.6)	318 (56.5)	258 (56.7)	0.94
BMI (kg/m^2^, mean + SD)	24.9 + 3.5	24.3 + 3.5	25.7 + 3.4	<0.001
BMI, <18.5, 18.5–24, 24–27, 27–30,>30 (kg/m^2^, %)	1.9%, 39.8%, 33.5%, 16.8%, 8.1%	3.2%, 46.5%, 29.8%, 14.6%, 5.9%	0.2%, 31.4%, 38.0%, 19.6%, 10.8%	<0.001
Diabetes, n (%)	158 (15.5)	72 (12.8)	86 (18.9)	0.007
Cholesterol (mg/dL, mean + SD)*	167 + 33	167 + 33	166 + 34	0.53
Triglyceride (mg/dL, mean + SD)*	103 + 57	109 + 64	96 + 49	0.004
Platelet count (x10^3^*u*/L, mean + SD)	164 + 58	165 + 56	162 + 59	0.44
AST (IU/L,mean + SD )	104 + 62	104 + 67	103 + 54	0.96
ALT (IU/L,mean + SD)	157 + 103	159 + 114	155 + 86	0.58
Ferritin(ng/ml, mean + SD)	406 + 472	400 + 554	415 + 344	0.61
*r*-GT (U/L,mean + SD)	67.2 + 63.3	62.3 + 62.5	73.4 + 63.7	0.005
HCV genotype 1, n/N (%) (N = 1012)	604/1012 (59.7)	344/562 (61.2)	260/450 (59.5)	0.27
HCV RNA (log IU/mL, mean + SD)	5.38 + 0.98	5.37 + 1.03	5.39 + 0.92	0.81
HBsAg(+), n (%)	90 (8.8)	53 (9.4)	37 (8.1)	0.47
Fibrosis 3-4, n (%)	308 (30.3)	162 (28.8)	146 (32.1)	0.25
*PNPLA3* rs738409 genotype				
CC/CG/GG, n (%)	422 (41.5)/ 477 46.9)/ 119 (11.7)	247 (43.9)/ 272 (48.3)/ 44 (7.8)	175 (38.5) / 205 (45.1)/75 (16.5)	<0.001
Recessive model GG, n (%)	119 (11.7)	44 (7.8)	75 (16.5)	<0.001
Dominant model GG+GC, n (%)	596 (58.5)	316 (56.1)	280 (61.5)	0.08

Note:

^*^data available in 694 patients. BMI: body mass index; *r*GT: *r-*glutamyltransferase; AST: alanine aminotransferase; ALT: aspartate aminotransferase; HBsAg: hepatitis B surface antigen; *PNPLA3*: patatin-like phospholipase domain-containing 3.

**Table 2:** Logistic regression analysis of factors associated with hepatic steatosis.

Variables	OR	95% C.I.	*P* value
Model 1 (without TG as variable, n = 1018)			
Recessive Model			
Age			
Per 1 year increase	1.02	1.004–1.028	0.007
BMI			
Per 1 kg/m^2^increase	1.12	1.082–1.167	<0.001
PNPLA3 rs738409 genotype			
CC/CG	1		
GG	2.34	1.557–3.515	<0.001
Dominant Model			
Age			
Per 1 year increase	1.02	1.004–1.028	0.006
BMI			
Per 1 kg/m^2^increase	1.13	1.084–1.169	<0.001
PNPLA3 rs738409 genotype			
CC	1		
GG+GC	1.31	1.013–1.703	0.04
Model 2 (with triglyceride as variable, n = 694)			
Recessive Model			
Age			
Per 1 year increase	1.02	1.009–1.038	0.001
BMI			
Per 1 kg/m^2^increase	1.09	1.043–1.140	<0.001
PNPLA3 rs738409 genotype			
CC/CG	1		
GG	2.37	1.408–3.983	0.001
Triglyceride	1		
Per 1 mg/dL increase	1.004	1.001–1.007	0.02
Dominant Model			
Age			
Per 1 year increase	1.02	1.009–1.038	0.001
BMI			
Per 1 kg/m^2^increase	1.09	1.042–1.138	<0.001
Triglyceride	1		
Per 1 mg/dL increase	1.004	1.001-1.007	0.01

Note: OR: odds ratio; C.I.: confidence intervals; BMI: body mass index. *PNPLA3*: patatin-like phospholipase domain-containing 3.

**Table 3:** Univariate analysis of factors associated with hepatic steatosis stratified by body mass index.

	BMI<24 kg/m^2^			BMI24-27 kg/m^2^			BMI>27 kg/m^2^		
	Steatosis (-) (n = 282)	Steatosis (+) (n = 144)	P value	Steatosis (–) (n = 166)	Steatosis (+) (n = 173)	P value	Steatosis (–) (n = 115)	Steatosis (+) (n = 138)	P value
Age (years, mean + SD)	50.4 + 12.4	52.4 + 12.0	0.12	51.6 + 11.2	54.0 + 9.2	0.03	51.4 + 11.1	52.3 + 10.7	0.51
Male gender, n (%)	147 (52.1)	80 (55.6)	0.50	101 (60.8)	97 (56.1)	0.37	70 (60.9)	81 (58.7)	0.73
Diabetes, n (%)	33 (11.7)	18 (12.5)	0.81	16 (9.6)	35 (20.2)	0.006	23 (20.0)	33 (23.9)	0.46
Platelet count (x10^3^*u*/L, mean + SD)	166 + 56	161 + 57	0.39	170 + 56	158 + 56	0.04	155 + 55	169 + 65	0.07
GOT (IU/L,mean + SD )	105 + 66	101 + 50	0.51	104 + 70	104 + 56	0.98	102 + 63	106 + 57	0.56
GPT (IU/L,mean + SD)	160 + 112	152 + 76	0.43	160 + 115	154 + 97	0.61	155 + 119	160 + 83	0.69
Ferritin(ng/ml, mean + SD)	408 + 632	363 + 306	0.32	408 + 542	445 + 351	0.45	366 + 317	431 + 368	0.14
*r*-GT (U/L,mean + SD)	56.4 + 61.4	63.5 + 43.3	<0.001	65.4 + 60.6	74.1 + 58.4	0.035	72.2 + 66.4	82.9 + 83.6	0.04
HCV genotype 1, n/N (%)	170/282 (60.3)	80/142 (56.3)	0.44	107/166 (64.5)	95/172 (55.2)	0.08	67/114 (58.8)	85/136 (62.5)	0.55
HCV RNA(log IU/mL, mean + SD)	5.28 + 1.05	5.30 + 0.90	0.84	5.42 + 1.05	5.36 + 0.92	0.56	5.53 + 0.93	5.52 + 0.92	0.92
HBsAg (+), n (%)	33 (11.7)	8 (5.6)	0.04	14 (8.4)	15 (8.7)	0.94	6 (5.2)	14 (10.1)	0.15
F34, n (%)	66 (23.4)	38 (26.4)	0.50	46 (27.7)	61 (35.3)	0.14	50 (43.5)	47 (34.1)	0.13
PNPLA3 rs738409									
CC/CG/GG, n (%)	117 (41.5)/ 147 (52.1)/ 18 (6.4)	43 (29.9)/ 73 (50.7)/ 28 (19.4)	<0.001	74 (44.6)/ 78 (47.0)/ 14 (8.4)	75 (43.4)/72 (41.6)/26 (15.0)	0.16	56 (48.7)/ 47(40.9)/ 12 (10.4)	57 (41.3)/ 60 (43.5)/ 21 (15.2)	0.37
Recessive model GG, n (%)	18 (6.4)	28 (19.4)	<0.001	14 (8.4)	26 (15.0)	0.06	12(10.4)	21 (15.2)	0.26
Dominant model GG+GC, n (%)	165 (58.5)	101 (70.1)	0.02	92 (55.4)	98 (56.6)	0.82	59 (51.3)	81 (58.7)	0.24

Note: BMI: body mass index; *r*GT: *r-*glutamyltransferase; AST: alanine aminotransferase; ALT: aspartate aminotransferase; HBsAg: hepatitis B surface antigen; *PNPLA3: patatin-like phospholipase domain-containing 3.*

**Table 4:** Logistic regression analysis of factors associated with hepatic steatosis in patients with different BMI.

BMI	Variables	OR	95% C.I.	*P* value	
<24 kg/m^2^					
	Recessive Model				
	*PNPLA3* rs738409 genotype				
	CC/CG	1			
	GG	3.44	1.824–6.500	<0.001	
	Dominant Model				
	HBsAg				
	Negative	1			
	Positive	0.42	0.188–0.940	0.04	
	*PNPLA3* rs738409 genotype				
	CC	1			
	GG+GC	1.69	1.101–2.614	0.02	
24–27 kg/m2					
	Age				
	Per 1 year increase	1.023	1.001–1.045	0.04	
	Diabetes				
	No	1			
	Yes	2.201	1.055–3.875	0.03	

Note: OR: odds ratio; C.I.: confidence intervals; BMI: body mass index. *PNPLA3*: patatin-like phospholipase domain-containing 3; HBsAg: hepatitis B surface antigen.

**Table 5:** Interaction of *PNPLA*3 rs738409 genotype and body mass index in hepatic steatosis.

BMI (kg/m^2^) & *PNPLA3* rs738409 genotype	Steatosis (%)	OR	95% CI	Adjusted P value*	Trend P value
BMI<24/non-GG	30.5%	1		Ref	<0.001
BMI<24/GG or BMI>24/non-GG	51.8%	2.43	1.849-3.202	<0.001	
BMI>24/GG	63.5%	3.87	2.292-6.524	<0.001	

Note:

^*^adjust age and sex; BMI: body mass index
