# Supplementary material for: PNPLA3 genetic variants determine hepatic steatosis in non-obese chronic hepatitis C patients
Source: Sci Rep. 2015 Jul 3;5:11901. doi: 10.1038/srep11901 (PMC4490397; doi:10.1038/srep11901)

***PNPLA3* genetic variants determine hepatic steatosis in non-obese chronic hepatitis C patients**

Chung-Feng Huang, Jyh-Jou Chen, Ming-Lun Yeh, Ching-I Huang,[2]Ming-Yen Hsieh, Hua-Ling Yang, Chia-Yen Dai,
Jee-Fu Huang, Zu-Yau Lin, Shinn-Cherng Chen, Wan-Long Chuang,Yao-Li Chen, Ming-Lung Yu

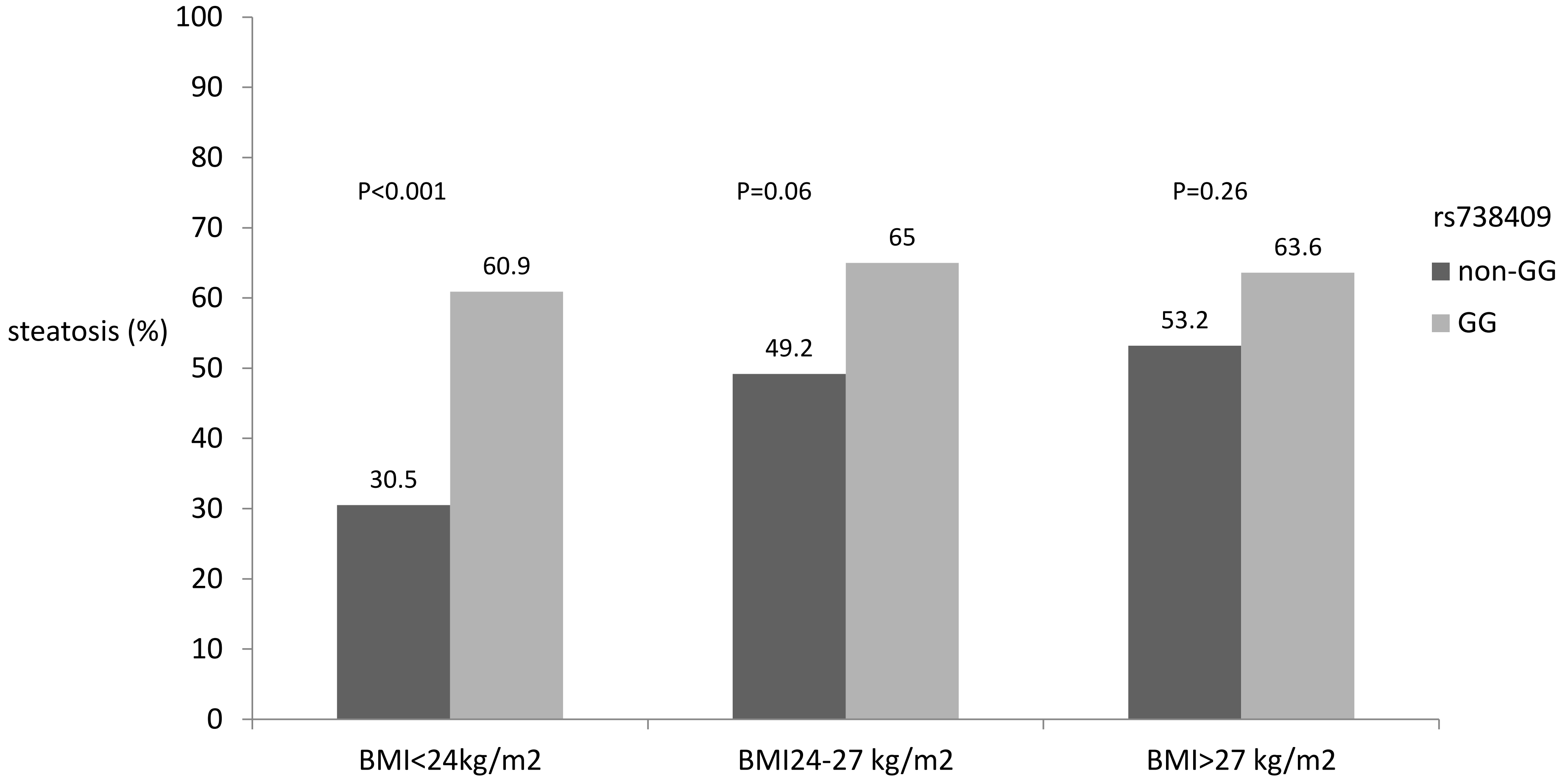

Supplement: Supplementary Information [file srep11901-s1.pdf]
